# High responsivity colloidal quantum dots phototransistors for low-dose near-infrared photodetection and image communication

**DOI:** 10.1038/s41377-025-01853-7

**Published:** 2025-05-19

**Authors:** Shijie Zhan, Benxuan Li, Tong Chen, Yudi Tu, Hong Ji, Diyar Mousa Othman, Mingfei Xiao, Renjun Liu, Zuhong Zhang, Ying Tang, Wenlong Ming, Meng Li, Hang Zhou, Bo Hou

**Affiliations:** 1 School of Physics and Astronomy, Cardiff University, The Parade, Cardiff, CF24 3AA UK; 2 International Collaborative Laboratory of 2D Materials for Optoelectronics Science and Technology of Ministry of Education, Institute of Microscale Optoelectronics, Shenzhen University, Shenzhen, 518060 China; 3 School of Electronic and Computer Engineering, Peking University Shenzhen Graduate School, Shenzhen, 518055 China; 4 Department of Instruments Science and Technology, School of Mechanical Science and Engineering, Huazhong University of Science and Technology, Wuhan, 430074 China; 5 Key Lab for Special Functional Materials of Ministry of Education, National and Local Joint Engineering Research Center for High-Efficiency Display and Lighting Technology, School of Nanoscience and Materials Engineering, Collaborative Innovation Center of Nano Functional Materials and Applications, Henan University, Kaifeng, 475004 China; 6 School of Engineering, Cardiff University, The Parade, Cardiff, CF24 3AA UK

**Keywords:** Photonic devices, Quantum dots

## Abstract

The surging demand and adoption of infrared photodetectors (IRPDs) in sectors of imaging, mobile, healthcare, automobiles, and optical communication are hindered by the prohibitive costs of traditional IRPD materials such as InGaAs and HgCdTe. Quantum dots (QDs), especially lead chalcogenide (PbS) QDs, represent the next-generation low-bandgap semiconductors for near-infrared (NIR) detection due to their high optical absorption coefficient, tunable bandgap, low fabrication costs, and device compatibility. Innovative techniques such as ligand exchange processes have been proposed to boost the performance of PbS QDs photodetectors, mostly using short ligands like 1,2-ethanedithiol (EDT) and tetrabutylammonium iodide (TBAI). Our study explores the use of long-chain dithiol ligands to enhance the responsivity of PbS QDs/InGaZnO phototransistors. Long-chain dithiol ligands are found to suppress horizontal electron transport/leakage and electron trapping, which is beneficial for responsivity. Utilizing a novel ligand-exchange technique with 1,10-decanedithiol (DDT), we develop high-performance hybrid phototransistors with detectivity exceeding 10^14^ Jones. Based on these phototransistors, we demonstrate image communication through a NIR optical communication system. The long-ligand PbS QDs/InGaZnO hybrid phototransistor demonstrates significant potential for NIR low-dose imaging and optical communication, particularly in scenarios requiring the detection of weak light signals at low frequencies.

## Abstract (web summary)

Leveraging long-chain dithiol ligand exchange, active image sensors based on PbS colloidal quantum dots enable near-infrared low-dose image sensing and image communication with detectivity exceeding 10^14^ Jones.

## Graphical abstract

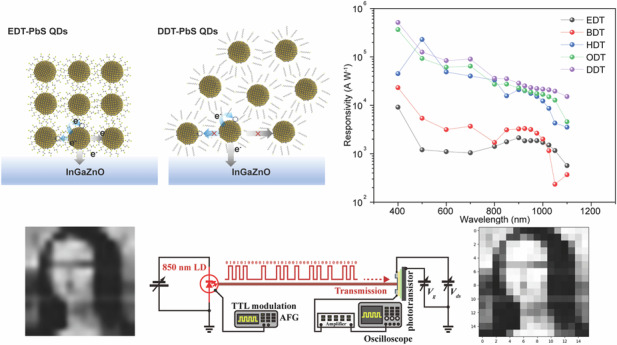

## Introduction

In recent years, there has been a growing demand for infrared photodetectors (IRPDs) across various sectors, including infrared imaging^1–3^, mobile and healthcare devices^4^, automobiles^5^, and optical communication^6^. This spectral region offers safe ocular exposure and efficient optical transmission in the atmosphere^2^. However, the complex fabrication processes and high costs of materials like InGaAs and HgCdTe in commercial IRPDs, significantly restrict their broader utilization^7^. Alternatively, colloidal quantum dots (QDs) have attracted extensive attention as near-infrared (NIR) light absorber materials in photodetectors, due to their unique properties, such as high optical absorption coefficient in NIR region, size and bandgap tunability, low-cost solution-based processing and good device compatibility. These properties make them a promising alternative to traditional IRPD materials^8–10^.

Among different QDs, PbS QDs have been applied to photodetectors with various device configurations^11–13^. A complementary metal-oxide-semiconductor (CMOS) image sensor array was fabricated by integrating PbS QDs and chemical vapor deposition (CVD)-grown graphene with CMOS readout circuits^14^. To date, 0D QDs/2D van der Waals nanosheet hybrid photodetectors have been realized^15,16^. In contrast to conventional CMOS image sensors, a flat-panel type imager can be manufactured on large glass or plastic substrates, opening possibilities for emerging large-scale imaging applications. Amorphous InGaZnO (IGZO) thin film transistors (TFTs), widely used as pixel drivers for flat-panel displays, could be integrated with PbS QDs photodetectors in flat-panel imaging optoelectronics^17,18^.

To address the issues of low photogenerated current and slow response in PbS QDs photodetectors, novel approaches have been proposed, including ligand exchange processes, doping, and the formation of heterojunctions. The initial ligands used in PbS QDs synthesis through thermal injection are long-chain oleic acid (OA), which hinders carrier transport between dots. To optimize surface ligands, both solid-phase and liquid-phase ligand exchange methods have become essential in developing QDs-based photodetectors. These processes involve replacing long-chain ligands with shorter ones, such as 1,2-ethanedithiol (EDT) and tetrabutylammonium iodide (TBAI). Over the past several years, a great amount of surface ligand engineering has been done on colloidal QDs to enhance responsivity and response speed by modifying the energy level of QDs and altering the interdot charge transport properties^19–21^. Among many studies, short ligands, including EDT and TBAI, provide relatively high effective mobility for PbS QDs and high photodetector performance^11,22^. The state-of-the-art photodetectors could obtain high detectivity over 10^12^ Jones and 3dB-bandwidth over 100 kHz based on short ligands^23^. However, the use of shorter ligands in exchange leads to issues such as film peeling and carrier quenching via surface traps^24^. Furthermore, due to the inherent trade-off between responsivity and response speed, QDs photodetectors fabricated through short ligand exchange cannot achieve ultrahigh responsivity, which limits their application in scenarios requiring the detection of extremely low-dose light signals^25^.

In this work, we adopted an innovative long ligand exchange approach and revealed the ability of 1,10-decanedithiol (DDT) to reach much higher responsivity in phototransistors compared to short ligands. Our work presents a responsivity-frequency transition for ligand-controlled photo-sensing on the platform of PbS QDs/IGZO phototransistors. Through characterization, including dark current and space charge limited current measurement, grazing incidence X-ray diffraction, photoluminescence quenching, and photothermal deflection spectroscopy, we found that long-chain alkanedithiol ligands improve phototransistor responsivity by suppressing horizontal transport and electron trapping in QDs matrix while enhancing vertical charge injection at the QDs/IGZO interface. By adopting DDT in ligand exchange, phototransistors with detectivity over 10^14^ Jones were achieved. Finally, the image transmission was successfully achieved through a NIR optical communication system using phototransistors.

## Results

### Responsivity enhancement in PbS QDs/IGZO phototransistors with long alkanedithiol ligands

Long alkyl chain molecules, such as 1-dodecanethiol, oleylamine, or oleic acid, are crucial in QDs synthesis. As ligands, their primary role involves passivating surface dangling bonds of QDs and stabilizing them to prevent aggregation in solvents. However, these long alkyl chains hinder carrier transport, resulting in low carrier mobility or even insulation in QD film. Therefore, converting these long molecular chains into short ligands is critical for optoelectronic devices. In 2006, Konstantatos et al. reported a photodetector made by exchanging 2.5 nm oleic acid (OA) with 0.6 nm *n*-butylamine, leading to increased carrier mobility and suppressed dark current^26^. Following a process of continuous updating and refinement, organic short ligands have transitioned to 3-mercaptopropionic acid (MPA)^27,28^, 1,2-ethanedithiol (EDT)^29,30^, 1,4-butanedithiol (BDT)^31,32^, etc.

**Fig. 1:**
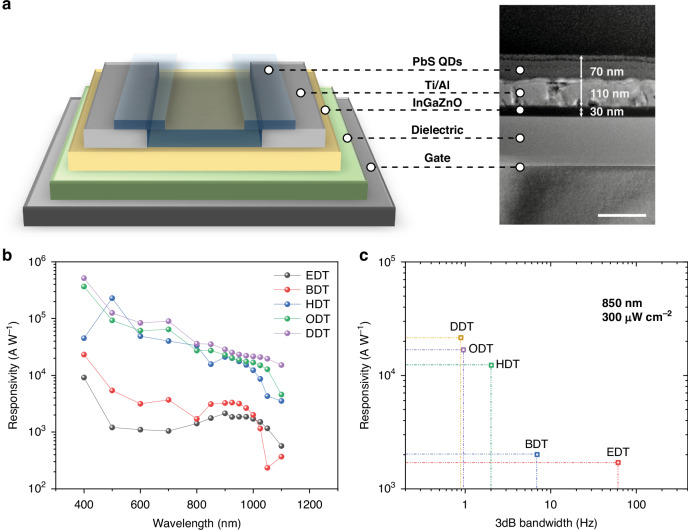
**Device structure and electro-optical characterization. a** Device diagram of the PbS QDs/IGZO phototransistor, and corresponding cross-sectional TEM image. The scale bar is 200 nm. Here, the device film structure is PbS QDs (70 nm)/Al (100 nm)/Ti (10 nm)/IGZO (30 nm)/SiO_2_ (200 nm) with Si substrate as gates. **b** Responsivity-wavelength plot of phototransistors with PbS QDs passivated by different length alkanedithiol ligands, varying from EDT to DDT. **c** Responsivity-bandwidth plot for phototransistors passivated with different length alkanedithiol ligands. Both responsivity measurements are conducted with *V*_*gs*_ = 5 V and *V*_*ds*_ = 5 V

In contrast to the conventional idea that short ligands give higher performance, we found that longer alkanedithiol molecules could provide PbS QDs with higher responsivity in PbS QDs/IGZO phototransistors. Figure 1a exhibits the device diagram where the IGZO channel was covered by PbS QDs as the light absorber of the hybrid phototransistor. The corresponding cross-section transmission electron microscopy (TEM) image of the PbS QDs/IGZO phototransistor is also shown. The thickness of IGZO/Ti/Al/PbS QDs is 30/10/100/70 nm, respectively (device structure and fabrication details are provided in Materials and Methods). Figure S1 shows the scanning electron microscope (SEM) image of the hybrid phototransistor. Alkanedithiol ligands (Table S1) with sulfhydryl at both ends and various carbons were adopted to passivate the PbS QDs, including EDT, BDT, 1,6-hexanedithiol (HDT), 1,8-octanedithiol (ODT), DDT. Figure S2 shows the Fourier-transform infrared spectroscopy (FTIR) spectrum and 2D FTIR mapping of PbS QDs film exchanged by these ligands to ensure the homogeneity of ligand coverage on QDs. Figure S3 presents the UV-visible absorption where the DDT peak has a low full-width half maximum, showing good packing homogeneity. The wavelength-dependent responsivity spectra of PbS QDs/IGZO phototransistors with different ligands are presented in Fig. 1b. It shows a peak wavelength between 800 and 1050 nm and a slight redshift for devices with shorter ligands, such as EDT, which is consistent with the UV–vis absorption spectra of PbS QDs (Fig. S3). With the increase of ligand length from EDT to DDT, the saturated responsivity gradually increases over the whole visible and near-infrared spectra. On the contrary, this causes an obvious trade-off between light responsivity and response speed, as shown in Fig. 1c. Figure S4 shows that the photoresponse of PbS QDs/IGZO phototransistors versus the response speed of incident light for different ligands, proving that short ligands enable high response speed due to reduced carrier transport time. Though DDT passivated PbS QDs/IGZO phototransistor has a relatively slower response compared to short ligand devices, its high responsivity over 2 × 10^4^ A W^-1^ is superior for low-dose light detection.

### Electron transport characterization to reveal the enhancement using long alkanedithiol ligands

**Fig. 2:**
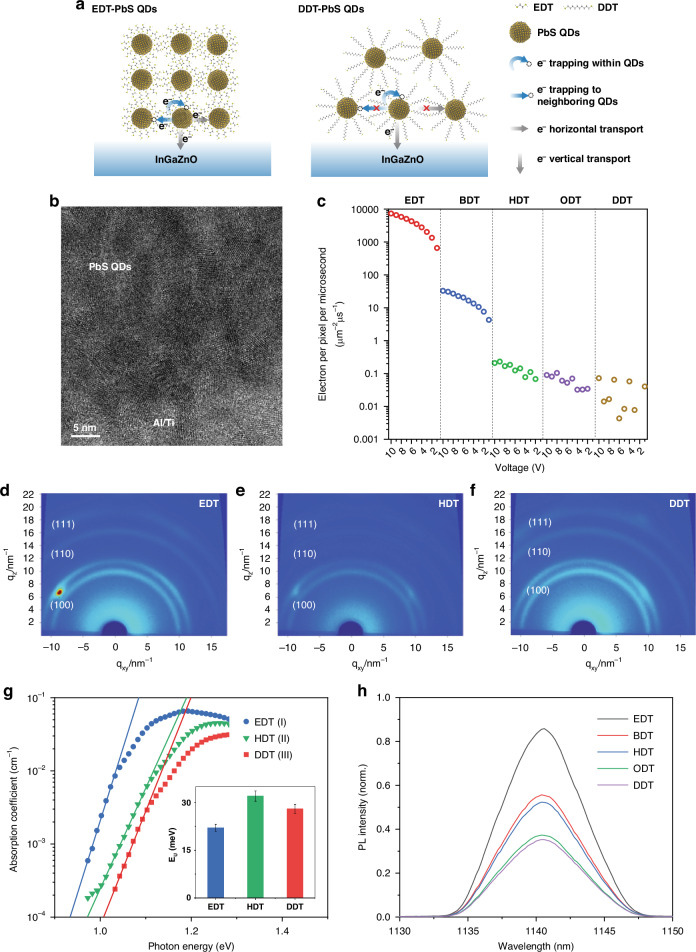
**Carrier transport path characterization of PbS-QDs films passivated by different ligands. a** The graphic summary illustrating the difference of charge transport between PbS QDs/IGZO films with EDT and DDT ligands, including electron trapping within QDs and to neighboring QDs, horizontal transport, and vertical transport to IGZO. **b** the TEM cross-section of PbS QDs. The scale bar is 5 nm. **c** The horizontal transporting electron density under dark circumstances at *V*_*ds*_ = 5 V. **d**–**f** GIXRD image for PbS QDs films passivated by EDT, HDT, and DDT. **g** PDS of EDT-PbS QDs, HDT-PbS QDs and ODT-PbS QDs. The insert image shows the Urbach-energy of PbS QDs films passivated by EDT, HDT, and DDT. **h** PL quenching for PbS QDs films treated by different ligands on IGZO film

Such special responsivity for long alkanedithiol could be induced by the competition of different electron transport paths within ligand-passivated PbS QDs. Figure 2a gives a graphic summary of different carrier transport paths in PbS QDs/IGZO films passivated by EDT and DDT. A high-resolution TEM cross-section image of PbS QDs is shown in Fig. 2b. The excited electrons in PbS QDs have three pathways: horizontal inter-dot transport, vertical transport to the IGZO channel, and electron trapping (within QDs and to neighboring QDs). Compared to the EDT films, horizontal leakage is also orders of magnitude lower in DDT films (Fig. 2c). Besides, the electron trapping paths to neighboring QDs are suppressed due to the long DDT chain length. The less electron trapping and horizontal leakage both result in better vertical electron transport to IGZO, which accounts for better light responsivity in DDT-QDs devices. The horizontal inter-dot transport is measured by the dark current of the PbS QDs conductor with Al as contact (device structure and fabrication in Materials and Methods), as shown in Fig. S5. The conductance of PbS QDs passivated by short ligands, which is 17 nS for EDT and 91 pS for BDT, is several magnitudes higher than the conductance of QDs passivated by longer ligands (<<1 pS). It is credited to the increased tunneling rate within the QDs matrix passivated by shorter ligands such as EDT^33^. Figure 2c extracts the horizontal transporting electron density from 0 V to 10 V of PbS QDs from the dark current data. For devices with short ligands, the large horizontal transport of over 3500 electrons per μm^2^ per μs for EDT-PbS QDs (at 5 V) shunts the excited electrons and suppresses the total responsivity of the phototransistors. As the ligand length increased, the tunneling rate of transport decreased and the PbS QDs mobility exponentially dropped^33^, which remarkably suppresses the horizontal carrier transport and reduces the horizontal leakage electron by over 100 times (compared to BDT), where not a single electron could be transported within QDs matrix passivated by HDT/ODT/DDT per μm^2^ per μs.

The horizontal transport of PbS QDs with different-length ligands was further characterized through QDs orientation through grazing incidence X-ray diffraction (GIXRD). Figure 2d–f and Fig. S6 show the 2D diffraction pattern of PbS QDs films passivated by alkanedithiol ligands with anisotropic intensity distribution in different azimuthal angles (*φ*). The asymmetric pattern, especially the strong (100) peak spot observed in EDT-PbS QDs thin films, might be related to the oriented assembly of QDs, as reported in previous works^34,35^. Typically, after ligand treatment (from EDT to DDT), PbS QDs tend to assemble along {100} direction^35,36^. However, compared with the 2D diffraction pattern of PbS QDs films passivated by HDT and DDT, the EDT-PbS QDs sample exhibits a stronger peak intensity of (100) facet at *φ* ~ 40.5°. This is consistent with previously reported textured EDT-PbS QDs film, and such EDT-PbS QDs film could result in large horizontal leakage current^35^. The PbS QDs films treated with HDT and DDT exhibit much more homogeneous intensity distribution over the azimuthal angle (Fig. 2e, f). Such a phenomenon may be caused by the less energy-preferred exchange of DDT due to its much longer chain length over EDT^36^. It should be noted that QDs diameter and distance among the neighboring QDs also influence the orientation of the assembly^35^. The PbS QDs used in this work are around 4 nm, which is similar to the reported EDT-bridged PbS QDs assembly (4.5 nm) exhibiting a rhombic lattice^35^. In addition, the oleic acid and EDT-coated PbS QDs also showed similar asymmetric bright GIXRD spots in previously reported PbS QDs assembly^35^ and systematic TEM studies^36^.

In addition to less electron shunt in the horizontal transport path, more vertical transport to the IGZO channel and less electron trapping contribute to the high responsivity of PbS QDs passivated by long alkanedithiol ligands. Interdot distance variation of different ligands impacts the electronic states in QDs solids, including the tail states under the conduction band. These serve as trapping states for excited electrons in QDs. The measurements of the photothermal deflection spectroscopy (PDS) (Fig. 2g) indicate that the Urbach-energy of DDT-PbS QDs is only 27.5 meV, which is even lower than that of HDT-PbS QDs ~ 32.5 meV, as well as BDT-PbS QDs and ODT-PbS QDs (Fig. S7). Thus, it can be inferred that excited electrons captured by tail states in DDT-PbS QDs are limited compared to QDs with shorter ligands. Although the PDS characterization shows that EDT-PbS and DDT-PbS QDs films have comparable tail states, the short distance between EDT-PbS QDs causes more electron trapping to neighboring QDs traps^24^, while in DDT-PbS QDs, this trapping process is suppressed due to long QDs distance, as the summary graph illustrated in Fig. 2a.

We further studied the vertical charge transport path at the PbS QDs/IGZO interface by measuring the photoluminescence (PL) of PbS QDs films on IGZO substrates (Fig. 2h). The PL intensity of PbS QDs/IGZO film decreases when the ligand chain is longer. As a reference, the PL intensity of PbS films shows an opposite trend^33^. This result infers that more excitons are quenched at the interface of IGZO from PbS QDs films passivated by DDT, indicating a better charge transport at the DDT-PbS QDs/IGZO interface. Better vertical transport is further supported by measuring the space charge limited current (SCLC) based on the device structure ITO/IGZO/PbS QDs/ZnO/Al (Fig. S8). The fitted effective mobility doesn’t show a simple trend according to chain length but shows efficient vertical electron transport for not only very short ligands such as EDT but also for long ligands such as DDT. It can be inferred that ligand orientation at the interface of PbS QDs/IGZO is potentially laid horizontally instead of standing vertically, inducing a more efficient electron transport in the vertical path than the horizontal one. Considering the horizontal transport, tail states trapping and vertical transport, maximum electron injection between PbS QDs and IGZO channel could be achieved by DDT exchange because of the highly suppressed horizontal inter-dot transport, limited tail states, and efficient vertical transport to IGZO channels.

### Reaching high-detectivity PbS QDs/IGZO phototransistors based on DDT

**Fig. 3:**
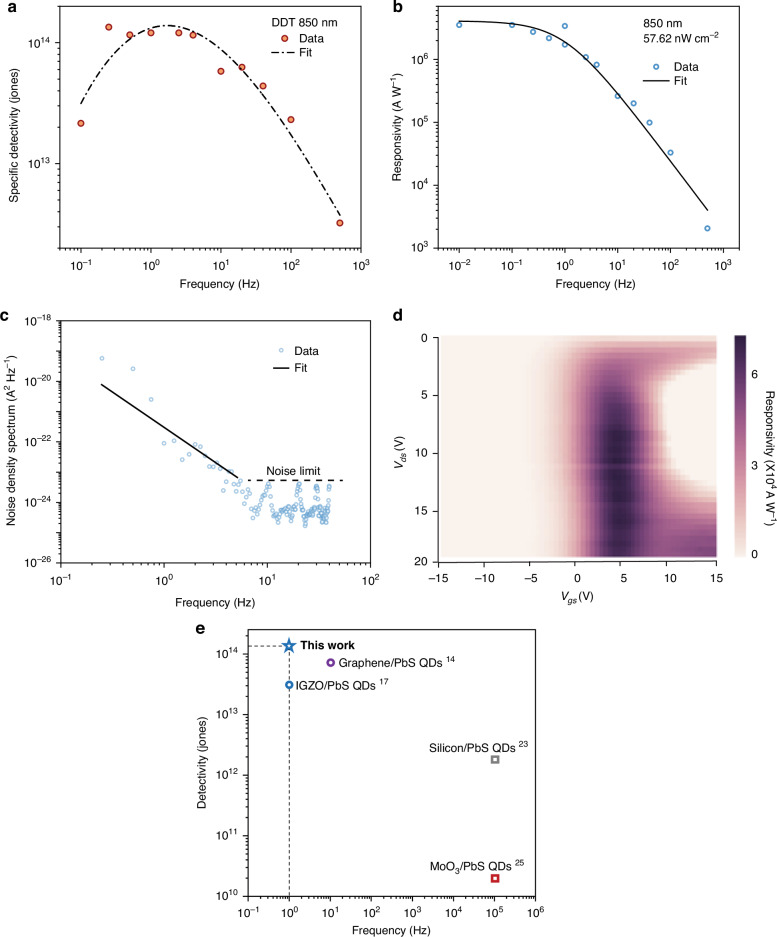
**Best electro-optic performance of DDT-PbS QDs/IGZO phototransistor with high detectivity. a**–**c** Frequency dependence of specified detectivity, responsivity, and noise density spectrum (*V*_*gs*_ = 5 V, *V*_*ds*_ = 15 V). **d** Voltage dependence of responsivity with incident power at 300 μW cm^-2^. The measurements are conducted with light at 850 nm. **e** Mapping of detectivity/frequency performance of the state-of-the-art PbS QDs phototransistors and the phototransistor in this work^14,17,23,25^

Based on the PbS QDs film passivated by DDT, the DDT phototransistors outperform devices with PbS QDs passivated by short ligands. We fabricated high performance DDT-PbS QDs/IGZO phototransistors that achieved specific detectivity over 10^14^ with frequency from 0.8 Hz to 3 Hz under the illumination of 57.62 nW cm^-2^ monochromic 850 nm light (Fig. 3a, see dark characteristics in Fig. S9) at room temperature (details in Materials and Methods). The frequency dependence of specific detectivity was calculated by responsivity and noise current at corresponding frequency^*^ (Fig. 3b, c)^37^:

$${D}^{* }=\sqrt{{Af}}R/{i}_{n}$$

The D*, *A*, *f*, *R*, *i*_*n*_ represent specific detectivity, effective device area, frequency, responsivity, noise current, respectively. The noise current of the PbS QDs/IGZO phototransistor shows 1/f noise component before the corner frequency ~ 8 Hz. The voltage dependence mapping of responsivity (Fig. 3d) indicates the preferred condition (*V*_*gs*_ ~ 5 V) to get around maximum responsivity, which is consistent with the mapping of IGZO TFT transconductance (Fig. S10). Both responsivity and transconductance rely more on gate voltage with drain voltage over 5 V. In addition to frequency-dependent responsivity variation, power-dependent photocurrent and responsivity are shown in Fig. S11. After incident light reaches 0.1 μW cm^-2^, the responsivity of the phototransistor starts to deteriorate, showing DDT-PbS QDs/IGZO phototransistor is highly sensitive under low dose incident light. The phototransistor in this work is compared with the state-of-the-art PbS QDs phototransistors, and the detectivity, frequency performance is summarized and depicted in Fig. 3e. More data is provided in Table S2.

### Demonstration of light communication based on long alkanedithiol PbS QDs/IGZO phototransistors

**Fig. 4:**
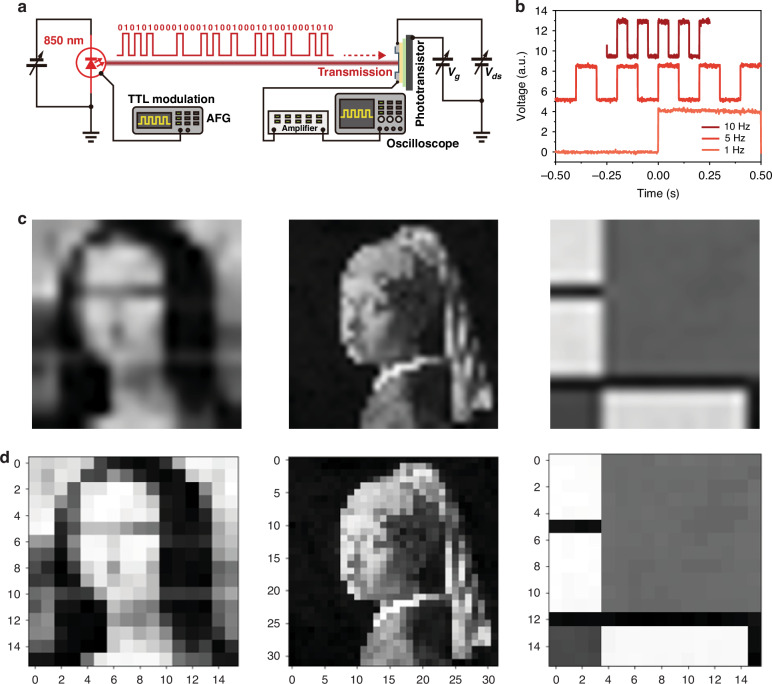
**Demonstration of the DDT-PbS QDs/IGZO phototransistor for applications in NIR optical communication. a** Schematic illustration of the NIR-LC system. The 850 nm NIR laser diode is modulated by an AFG. The NIR light signal transmits via free space and irradiates the sample to generate a photocurrent, which is amplified and recorded by an oscilloscope. When performing data transmission, the image pixel is encoded into 8-bit digital signals. The received signals are decoded by a computer. **b** Periodic photoresponse of the phototransistor under 850 nm laser light at frequencies of 1 Hz, 5 Hz and 10 Hz, respectively. **c** Original gray images and corresponding. **d** Received image by phototransistor

Finally, the potential of the hybrid PbS QDs/IGZO phototransistor as a fundamental component for an imaging system was evaluated through a NIR light communication (NIR-LC) system. As shown in Fig. 4a, a commercial NIR laser diode (LD) with a wavelength of 850 nm was used as a transmitter, and the DDT-PbS QDs/IGZO phototransistor was used as an optical signal receiver. The 850 nm LD was modulated by an arbitrary function generator (AFG) via transistor-transistor logic (TTL) signal control to realize on-off keying (OOK). The phototransistor received aligned laser light signals and transferred them into electrical signals, which were further amplified and recorded by an oscilloscope to evaluate the optoelectronic properties and communication performance. Figure 4b displays the transient photocurrent responses at *V*_*gs*_ = 20 V and *V*_*ds*_ = 15 V, measured at different modulation signal frequencies. It is clear that the DDT-PbS QDs/IGZO phototransistor can detect optical signals when the laser diode modulation frequency ranges from 1 Hz to 10 Hz. It is worth noting that the signal obtained in this NIR-LC system is voltage-based. Compared to current-based signals collected in the abovementioned responsivity-response speed measurements, the voltage-based signals in the NIR-LC system exhibit a slightly faster response ~ 10 Hz due to a reduced transconductance with a different gate bias. Here, the reduced transconductance also decreases the gain of the phototransistor but could boost the response speed to some degree due to a trade-off between photo-gain and response^38^. We further examine the communication performance of the DDT-PbS QDs/IGZO phototransistor for the NIR-LC system. The original grayscale images were digitally encoded according to the 8-bit grayscale of each pixel before communication. The encoded signals were then transmitted by the 850 nm LD by the OOK modulation. The received data was decoded by a control computer to reconstruct the images. Comparing Fig. 4e and f, three images were successfully transferred and received by our NIR-LC system.

## Discussion

In this report, it was found that for alkanedithiol ligands, long-chain ligands, though they have inferior frequency response, have superior responsivity. In the characterization of horizontal (dark current and GIXRD), vertical transport (SCLC and PL quenching), and tail states trapping (photothermal deflection spectroscopy), DDT-PbS QDs show suppressed horizontal electron transport and tail states trapping, as well as efficient vertical electron transport to IGZO channels.

Our research has focused on enhancing the performance of PbS QDs photodetectors by employing long-chain dithiol ligands. Traditional methods have utilized short ligands such as EDT and TBAI, which, while effective in certain aspects, do not fully optimize the potential of PbS QDs. Our approach with long-chain dithiol ligands, specifically DDT, has significantly improved the responsivity of PbS QDs/IGZO phototransistors with ultrahigh detectivity ~ 10^14^ Jones. While DDT-passivated phototransistors exhibit slower response speeds compared to those with short ligands, their high responsivity makes them suitable for low-dose light detection, which is essential in applications requiring high sensitivity but low dose. In addition, using long-chain ligands results in less electron shunting in the horizontal transport path and more efficient vertical transport. This is corroborated by photothermal deflection spectroscopy and photoluminescence quenching measurements, which indicate fewer trapping states and better charge transfer at the QDs/IGZO interface. In addition, we have investigated the device variation and 105 °C thermal-cycle stability in supporting information (Figs. S12 and S13) to show the good reproducibility and reliability of DDT- PbS QDs/IGZO phototransistors.

The practical application of these phototransistors in NIR-LC systems was successfully demonstrated. The ability to transmit and receive optical signals at various frequencies, including the successful transmission of gray-scale images, highlights the potential for these devices in real-world imaging and communication systems. This advancement paves the way for more cost-effective and efficient IRPDs in a variety of applications, addressing the limitations posed by traditional materials and fabrication techniques. Future works would focus on device and integrated module stability studies and developing a reset gate to reduce the fall time of PbS QDs/IGZO phototransistors to further decouple the trade-off between responsivity and response speed.

## Materials and methods

### Synthesis of PbS quantum dots

The PbS colloidal quantum dots synthesis was carried out following our previous works^36^. The Pb precursor was loaded in the two-neck flask 1 with 0.47 mg (2.1 mmol) PbO, which was dissolved in 2.25 ml of OA and 12.7 ml of 1-Octadecene (ODE), followed by degassing at 100 °C in vacuum for 3 h. The synthesis system was flushed with N_2_ and heated at 130 °C. Meanwhile, 210 µl of Tetramethylsilane (TMS) was dissolved in flask 2 with 4 mL of ODE, which was under degassing for 20 minutes in vacuum at 90 °C and then loaded in a syringe. The content of the syringe was injected swiftly into reaction flask 1, and the heating was removed once the injection was finished. Subsequently, flask 1 was left to cool down to room temperature by a water bath treatment. To remove unreacted reagents, the prepared mixture of QDs was first separated into two batches, and 30 ml of acetone was added to each batch to precipitate the PbS QDs. The suspension was then centrifuged at 7000 rpm for 10 min. 4 ml of hexane was subsequently added to the individual batch to re-dissolve the QDs, and 20 ml of methanol was further added to precipitate the QDs again. The resultant suspension was then centrifuged at 400 rpm for 4 min. The hexane/methanol washing process could be repeated once or twice to increase the purification of PbS QDs. The PbS QDs were finally dissolved in toluene and filled with N_2_, making a concentration of 50 mg ml^-1^ for further utilization.

### Device fabrication

The devices were fabricated on Si/SiO_2_ substrates or borosilicate glasses. Devices for comparing responsivity and frequency among different ligands were fabricated on Si/SiO_2_ substrates. The high performance DDT phototransistors were fabricated on borosilicate glass with patterned gates and high-k dielectric. Substrates were cleaned with acetone and 2-propanol in sonication for 10 min each, followed by N_2_ dry. For the Si/SiO_2_ substrate, the substrate was used as a gate/dielectric. For the borosilicate glass substrate, a patterned gate layer of 10 nm Ti/100 nm Al was deposited by an e-beam evaporator on the substrate. Then, a 50 nm AlO_x_ dielectric film was deposited by ALD at 150 °C. For the channel of devices, 30 nm IGZO (In:Ga:Zn=1:1:2 at%) film was sputtered on the wafer under room temperature with Ar flow at 25 sccm, pressure at 6.5 mtorr and power at 75 W. The IGZO channel was patterned and etched by 1% HCl solution. A top contact of 10 nm Ti/100 nm Al was deposited by an e-beam evaporator after photolithography processes. The device was annealed in ambient air at 300 °C for 1 h post-annealing. To form the PbS QDs light absorber on the IGZO channel, 1 drop of 50 mg ml^-1^ PbS QDs in hexane was spun at 2000 rpm for 30 s on the channel. Then, the PbS QDs film was treated in different dithiol ligand solutions (0.3 mM, acetonitrile) for 120 s at room temperature, followed by acetonitrile washing and N_2_ drying. Here, the room temperature ligand exchange was conducted to prevent acetonitrile from evaporating, causing excessive remaining ligand on QDs surface. In addition, the treatment time of 120 s was the longest time before the obvious peeling of QDs films to ensure sufficient ligand exchange time. The device was designed to have channel length and width of 20 μm and 720 μm.

### Responsivity and response measurement

The monochrome incident light was formed by a broadband light source and a set of optical filter. For the responsivity measurement, the photocurrent was measured using Keithley 4200 Semiconductor Analyzer under light sources with different wavelength. The phototransistors were biased with *V*_*gs*_ = 5 V and *V*_*ds*_ = 5 V.

For the bandwidth measurement, an 850 nm incident light at 300 μW cm^-2^ was modulated by a chopper with varying rotating speeds for various light frequencies at room temperature. The phototransistor current was measured by Keithley 4200 Semiconductor Analyzer. The phototransistors were biased with *V*_*gs*_ = 5 V and *V*_*ds*_ = 5 V. All the photoresponse was normalized to compare the response of different phototransistors (Fig. S4).

### Horizontal electron leakage current measurement

Planar Al-QDs-Al conductors were fabricated to measure the horizontal leakage current of PbS QDs passivated by different ligands. The channel length and width of the conductor were 10 μm and 1560 μm. The length was designed to be smaller than the real length of the phototransistor due to the low conductance of QDs. PbS QDs film deposition and ligand exchange followed the same process as the phototransistor. The leakage current was measured in dark condition with the voltage scanned from -10 V to 10 V.

The horizontal transported electron density was extracted from dark current data at *V*_*ds*_ = 5 V with transport cross-section area equals device width*QD thickness (around 1560 μm*0.1 μm).

### Electron only device fabrication and measurement

The electron-only devices were fabricated based on the structure as follows: ITO 100 nm/IGZO 30 nm/PbS QDs (toluene 50 mg ml^-1^ 2000 rpm cast)/ZnO nanoparticle 20 nm/Al 100 nm. The 20 nm ZnO nanoparticle was deposited by spin coating 25 mg ml^-1^ ZnO nanoparticle solution in ethanol at 4000 rpm for 30 s.

### Noise measurement

The preamplifier is connected in series with a phototransistor between the source and the ground. The output is connected to the channel of the Dynamic Signal Analyzer. Use the AC option, where the amplification gain can be chosen according to different current noise levels. The gain level was 10^-5^ A V^-1^ to match IGZO transistor dark current noise. The axis was set in the Log scale to show the flicker noise before the shot noise limit. The relationship between the signal in the analyzer and real noise is ${I}_{N}/{B}^{1/2}={\rm{Signa}}{{\rm{l}}}^{1/2}* {10}^{-5}$.

### NIR-LC system setup and measurement

A commercial 850 nm NIR LED with a 1 mW power was used as a transmitter, and the DDT-PbS QDs/IGZO phototransistor was used as an optical signal receiver. The laser diode was modulated by an AFG (RIGOL DG5252) via TTL signal. When performing data transmission, an on-off keying (OOK) scheme was used. The phototransistor was biased by the Keithley 4200 Semiconductor Analyzer. The output drain current signal of the phototransistor was input to the Stanford Research System Model SR570 low-noise current amplifier and transformed into voltage signals, which were recorded by Tektronix TDS2024C oscilloscope or decoded by a control computer.

## Supplementary information

Support Information

## Data Availability

Information on the data underpinning this publication, including access details, can be found in the Cardiff University Research Data Repository at: 10.17035/cardiff.28668266.
