# Supplementary material for: High responsivity colloidal quantum dots phototransistors for low-dose near-infrared photodetection and image communication
Source: Light Sci Appl. 2025 May 19;14:201. doi: 10.1038/s41377-025-01853-7 (PMC12086224; doi:10.1038/s41377-025-01853-7)
Supplement: Supplementary file 1 — Support Information [file 41377_2025_1853_MOESM1_ESM.docx]

Supporting Information

**High Responsivity Colloidal Quantum Dots Phototransistors for Low-Dose Near-Infrared Photodetection and Image Communication**

Shijie Zhan^1‡^, Benxuan Li^2‡^, Tong Chen^3^, Yudi Tu^2^, Hong Ji^1^, Diyar Mousa Othman^1^, Mingfei Xiao^4^, Renjun Liu^1^, Zuhong Zhang^5^, Ying Tang^5^, Wenlong Ming^6^, Meng Li^5*^, Hang Zhou^3*^ and Bo Hou^1*^

1. School of Physics and Astronomy, Cardiﬀ University, The Parade, Cardiﬀ CF24 3AA, UK *E-mail: [houb6@cardiff.ac.uk](mailto:houb6@cardiff.ac.uk)

2. International Collaborative Laboratory of 2D Materials for Optoelectronics Science and Technology of Ministry of Education, Institute of Microscale Optoelectronics, Shenzhen University, Shenzhen 518060, China

3. School of Electronic and Computer Engineering, Peking University Shenzhen Graduate School, Shenzhen 518055, China *E-mail: [zhouh81@pkusz.edu.cn](mailto:zhouh81@pkusz.edu.cn)

4. Department of Instruments Science and Technology, School of Mechanical Science and Engineering, Huazhong University of Science and Technology, Wuhan 430074, China

5. Key Lab for Special Functional Materials of Ministry of Education, National and Local Joint Engineering Research Center for High-Efficiency Display and Lighting Technology, School of Nanoscience and Materials Engineering, Collaborative Innovation Center of Nano Functional Materials and Applications, Henan University, Kaifeng 475004, China *E-mail: [mengli@henu.edu.cn](mailto:mengli@henu.edu.cn)

6. School of Engineering, Cardiff University, The Parade, Cardiff, CF24 3AA, UK

^‡^S. Zhan and ^‡^B. Li contributes equally.


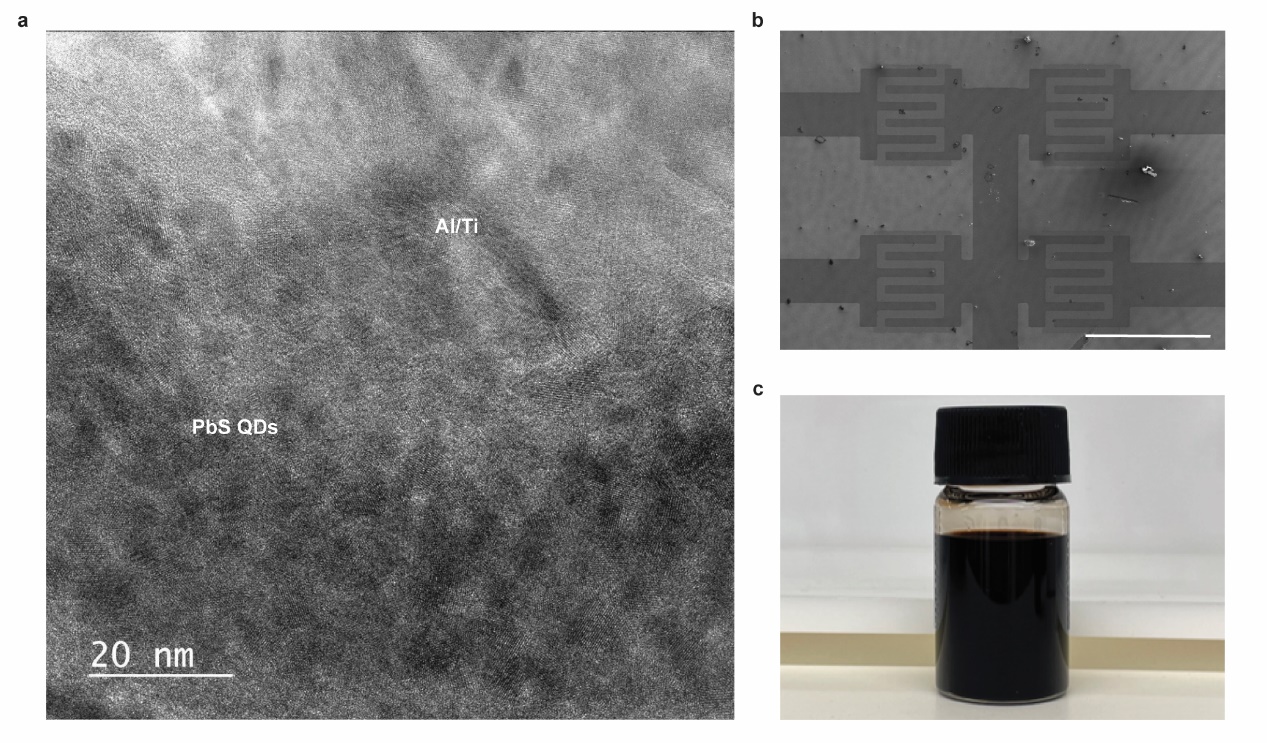


**Fig. S1 a** TEM lamella cross-sectional image of the QDs solids and interface and **b** the SEM image of the PbS QDs/InGaZnO (IGZO) phototransistor. The scale bar is 20 nm. The TEM lamella samples were prepared by a Helios Nanolab (FEI) SEM/focused ion beam (FIB). The scale bar is 300 μm. **c** As-prepared PbS colloidal QDs ink solution.

The transmission electron microscopy (TEM) image shows a clear interface between the Al/Ti electrode layer and the top PbS quantum dots (QDs) photo-sensing layer. The scanning electron microscopy (SEM) image shows the layout of the phototransistors with interdigital contacts. This also demonstrates the good dispersibility of the as-obtained PbS QDs and its well-attached interconnecting formed QDs solids, which are prerequisites for good device performance.

**Table. S1** List of the chemical formula and length^1^ of different alkanedithiol ligands used in this work.


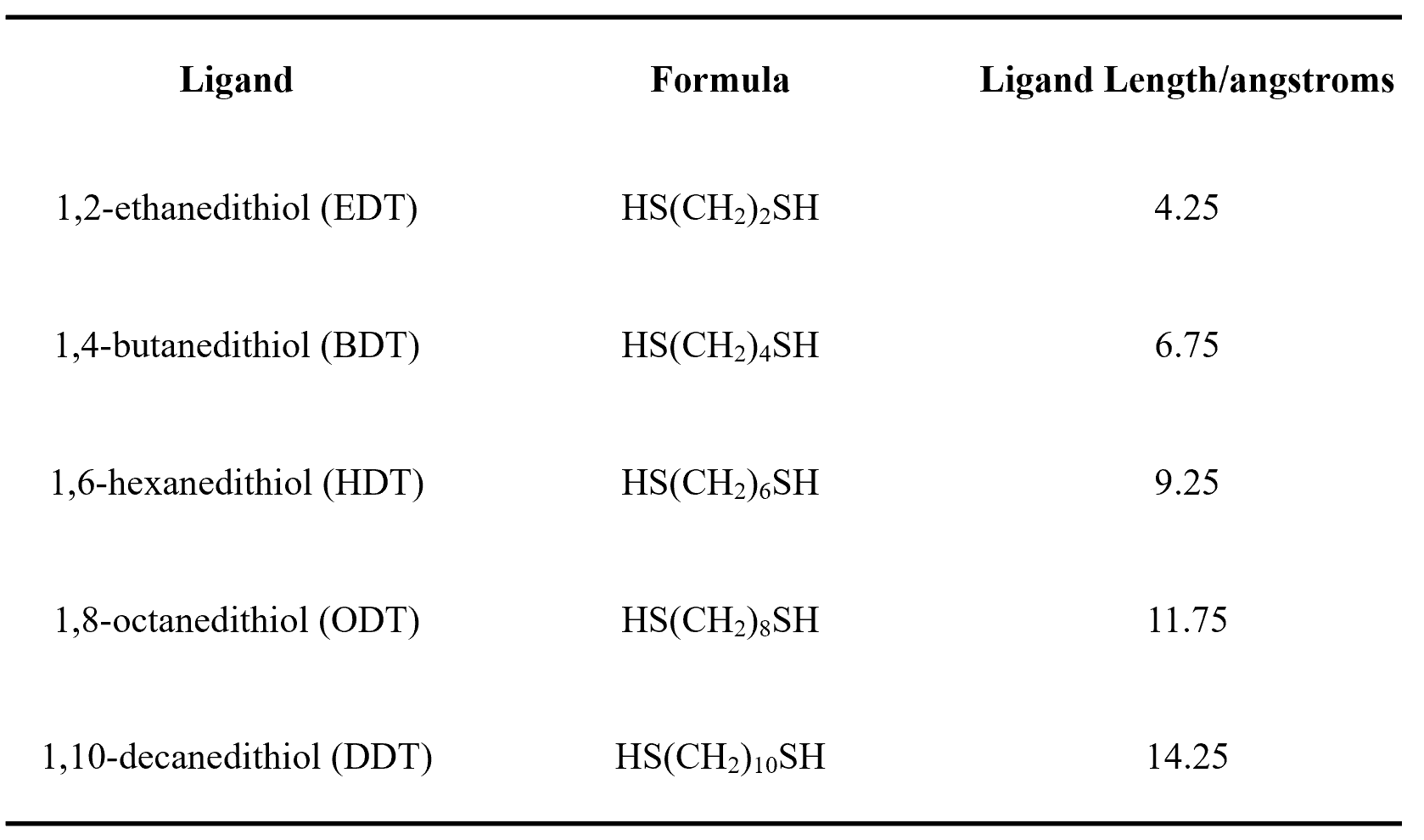


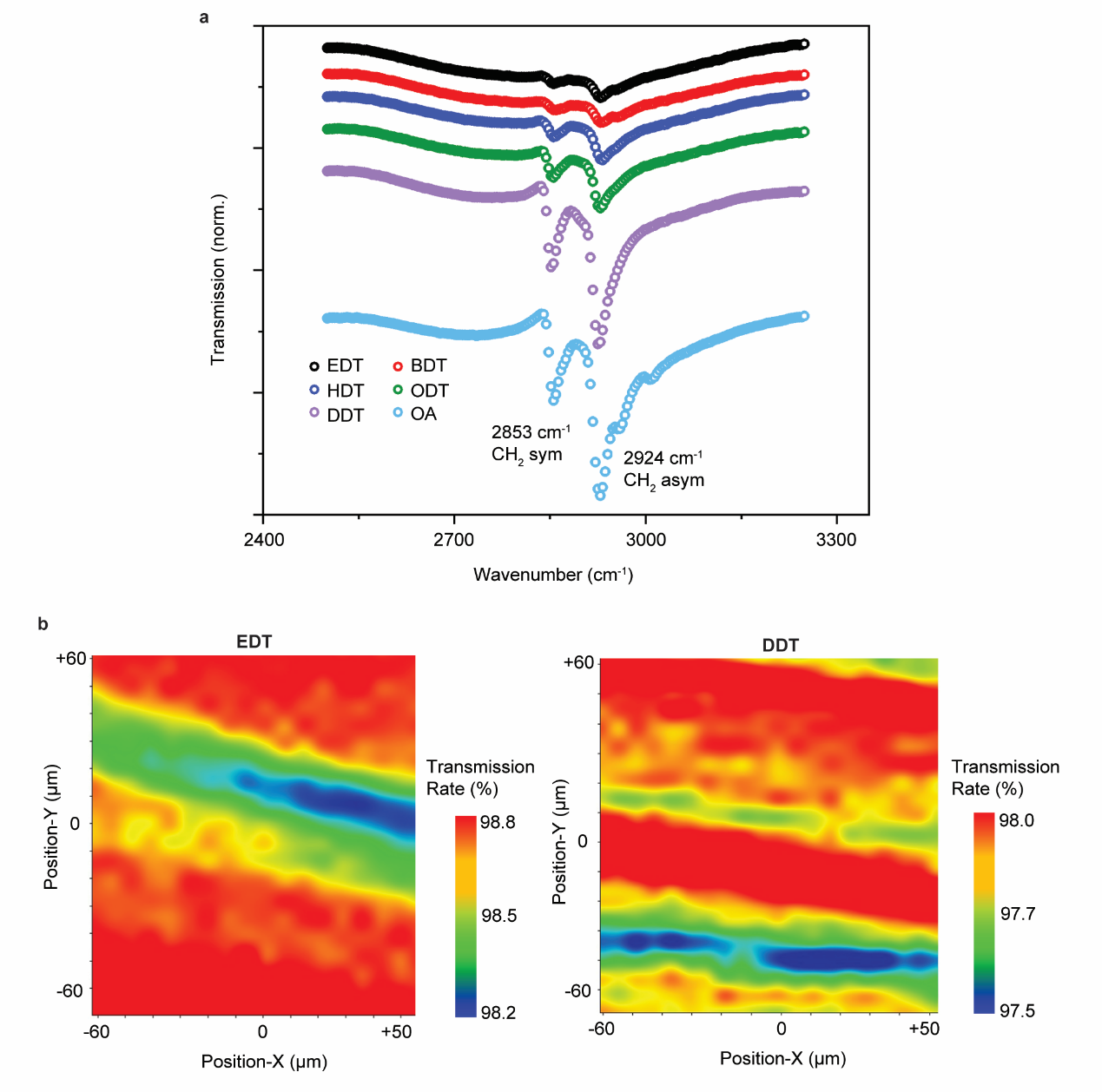


**Fig.** **S2** **a** The Fourier Transform Infrared (FTIR) spectrum of PbS QDs film exchanged by different length alkanedithiol ligands and oleic acid (OA). Here alkanedithiol ligands include 1,2-ethanedithiol (EDT), 1,4-butanedithiol (BDT), 1,6-hexanedithiol (HDT), 1,8-octanedithiol (ODT), 1,10-decanedithiol (DDT). **b** The 2D FTIR mapping of transmission intensity at 2853 cm^-1^ for EDT-PbS and DDT-PbS QDs films over an area of 110 μm*120 μm.

Dramatic reduction in the intensity of C-H vibrations at 2924 cm^-1^ (asymmetric) and 2853 cm^-1^ (symmetric) are signatures of the presence of OA. As the length of the alkane chain decreases between oleic acid and alkanedithiol ligands, the intensity of the CH_2_ pattern decreases with the reduction of ligand length. The transmission rate at 2853 cm^-1^ is measured using 2D FTIR mapping over a large film area of 110 μm*120 μm for EDT and DDT QDs films. The 2D FTIR mapping of both EDT-PbS QDs and DDT-PbS QDs show good uniformity with transmission rate variation within 0.3 % over 110 μm*120 μm area. It indicates that both short and long-ligand treatments on PbS QDs have good homogeneity and uniform coverage.


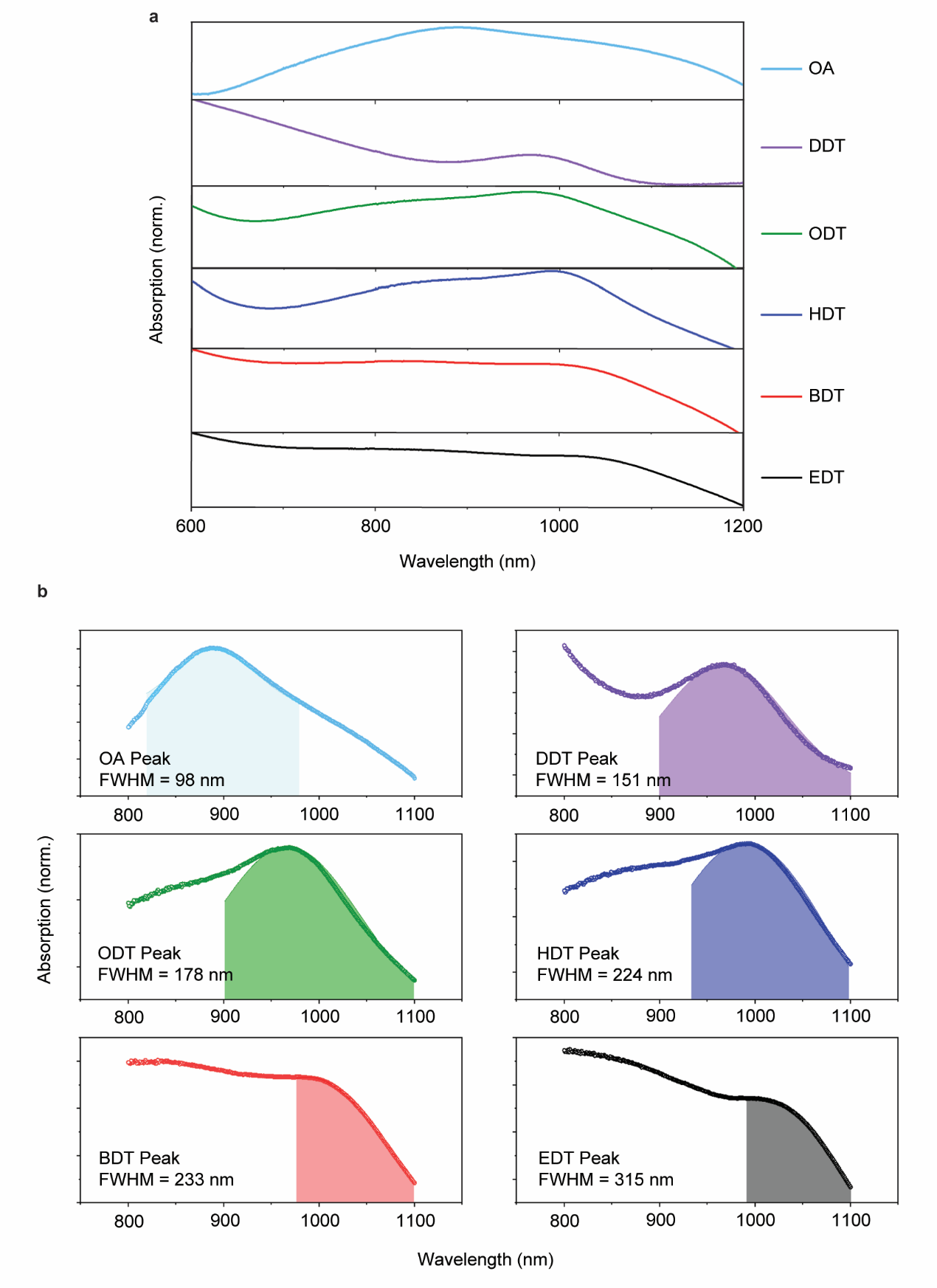


**Fig.** **S3** **a** The visible-infrared spectrum of PbS QDs film exchanged by different ligands. There is an absorption peak within 850~1000 nm, where the peak wavelength of PbS QDs is red-shifted with shorter ligand passivation. **b** The Gaussian fitting of the absorption peaks for QDs films passivated by different ligands. The full-width half maximum (FWHM) increases with the reduction of ligand length.

Additionally, as shown in Fig. S3a, the exciton absorption peaks were broadened and red-shifted. These changes can be attributed to electron coupling or sintering/necking between QDs as the inter-particle distance decreases ^2-4^. This observation aligns with the increased packing densities in the TEM analysis in Fig. S1.

Fig. S3b shows that the DDT-PbS QDs absorption curve has a sharp peak with lower FWHM compared to shorter ligands (EDT, BDT, HDT, ODT), showing that DDT-PbS QDs have good packing homogeneity and uniformity ^5^. This indicates that DDT ligands achieve a uniform passivation on PbS QDs.


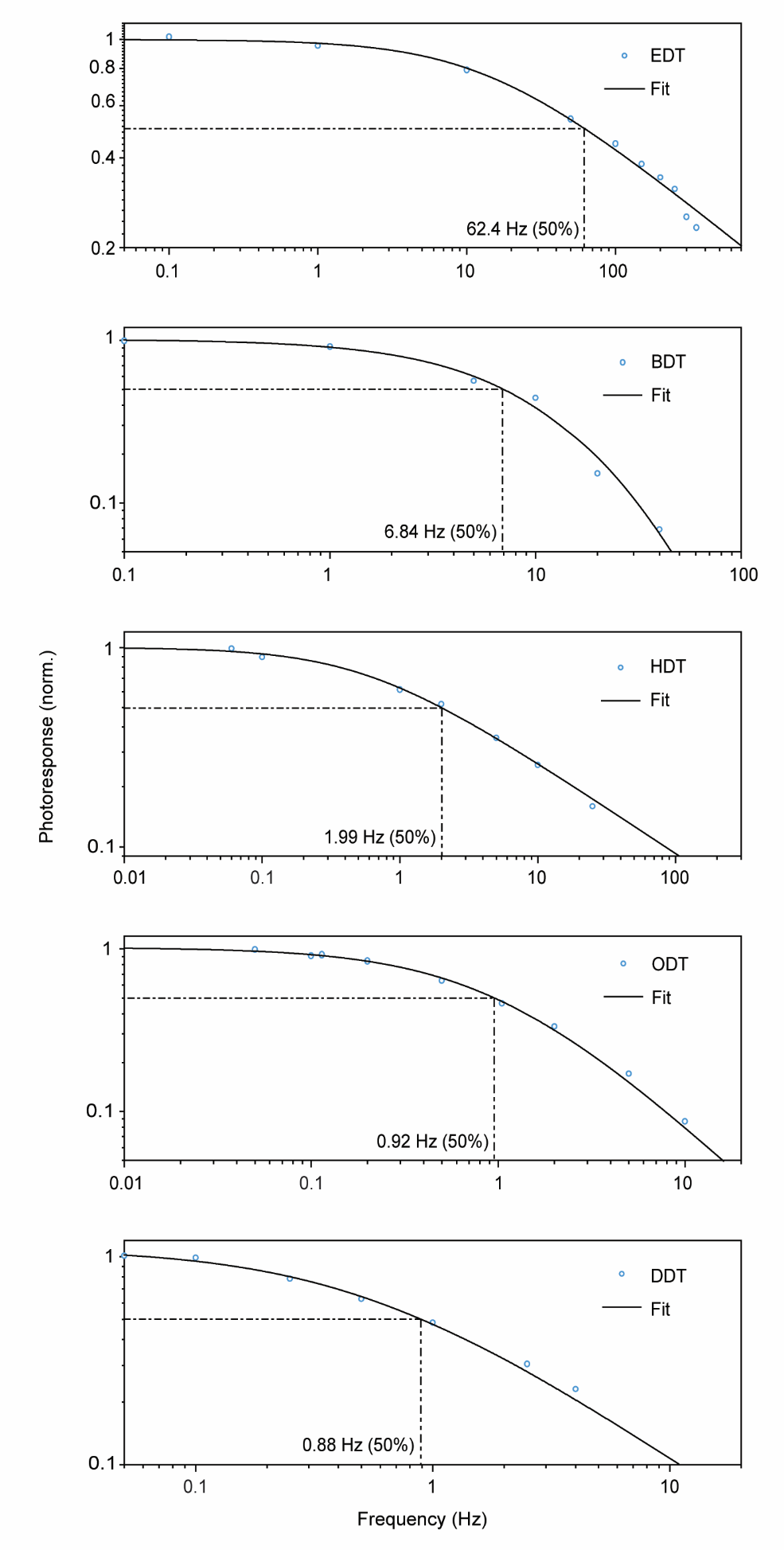


**Fig.** **S4** The normalized photoresponse of PbS QDs/IGZO phototransistors versus the frequency of incident light for different ligands.

Fig. S4 shows the speed response for PbS QDs/IGZO phototransistors with various ligands from EDT to DDT, characterized by the 4200 Keithley Semiconductor Analyzer. Generally, all phototransistors with different ligands have response speed degradation with the increase of light frequency. Here, it is evident that a shorter ligand provides the phototransistor with a faster response speed. The 3dB (50 % responsivity drop) frequency is 62.4 Hz, 6.84 Hz, 1.99 Hz, 0.92 Hz, and 0.88 Hz for phototransistors with ligand exchange of EDT, BDT, HDT, ODT, and DDT, respectively.


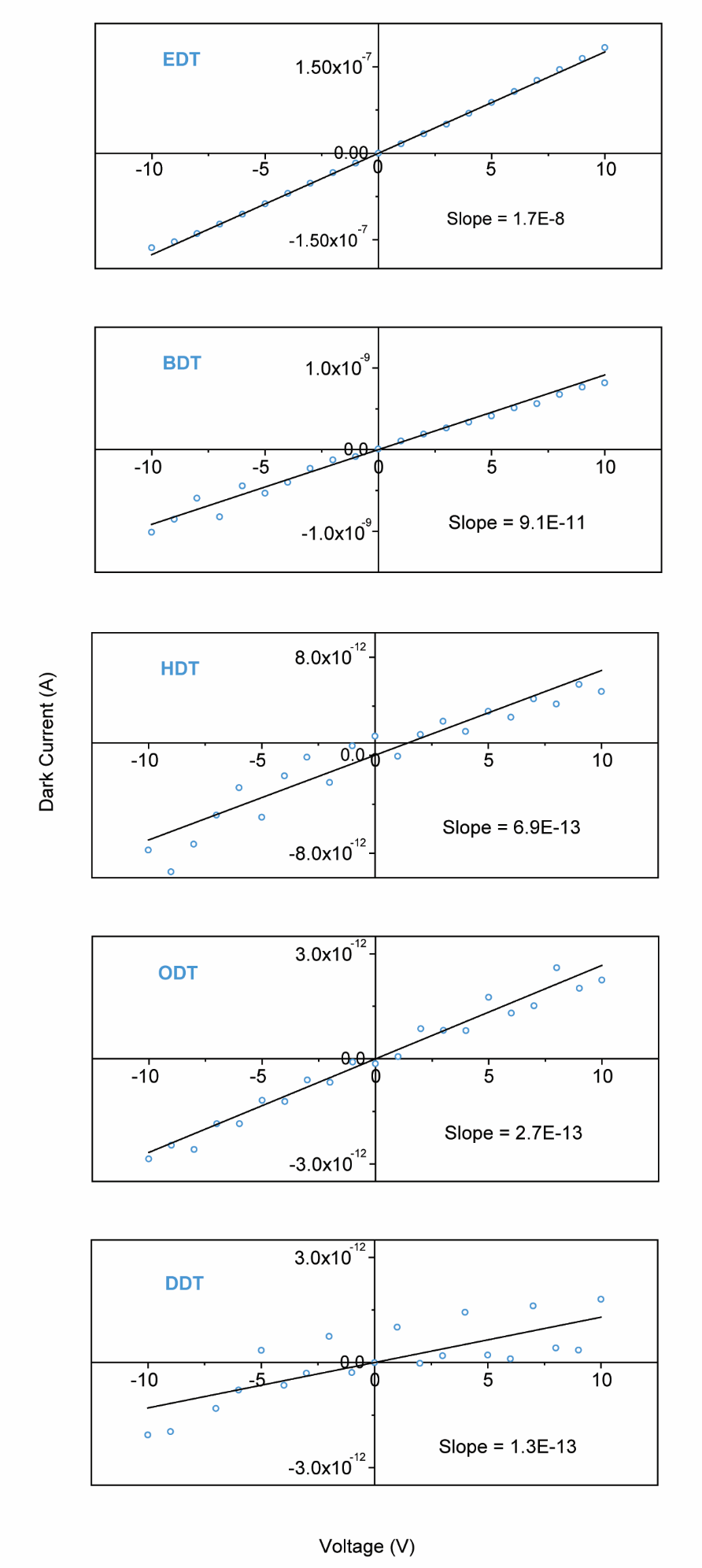


**Fig.** **S5** The dark current of the PbS QDs conductor with Al as the contact. PbS QDs films are exchanged with ligands from EDT to DDT.

The horizontal transport is studied by measuring dark current in planar Al-QDs-Al conductors with various ligands on PbS QDs, as shown in Fig. S5. The channel length and width of the conductor were 10 μm and 1560 μm. The leakage current was measured in dark conditions with the voltage scanned from -10 V to 10 V. Here, the slope of the *I-V* plot represents the conductivity of PbS QDs under specific ligand passivation. It is noticed that shorter ligands, including EDT and BDT, have over two orders higher conductivity compared to longer ligands, including HDT, ODT and DDT.

The reduced leakage current of long ligands, especially DDT, is mainly due to two reasons: 1) the longer ligand chain length results in a larger gap between PbS QDs, reducing mobility for charge transport ^1^; 2) The GIXRD shown in Fig. 2d-f and Fig. S6 indicate a more preferred assembly in shorter alkanedithiol ligands, especially EDT. The well-oriented QDs packing results in better charge transport and higher leakage current in QDs film passivated with shorter ligands ^6^.


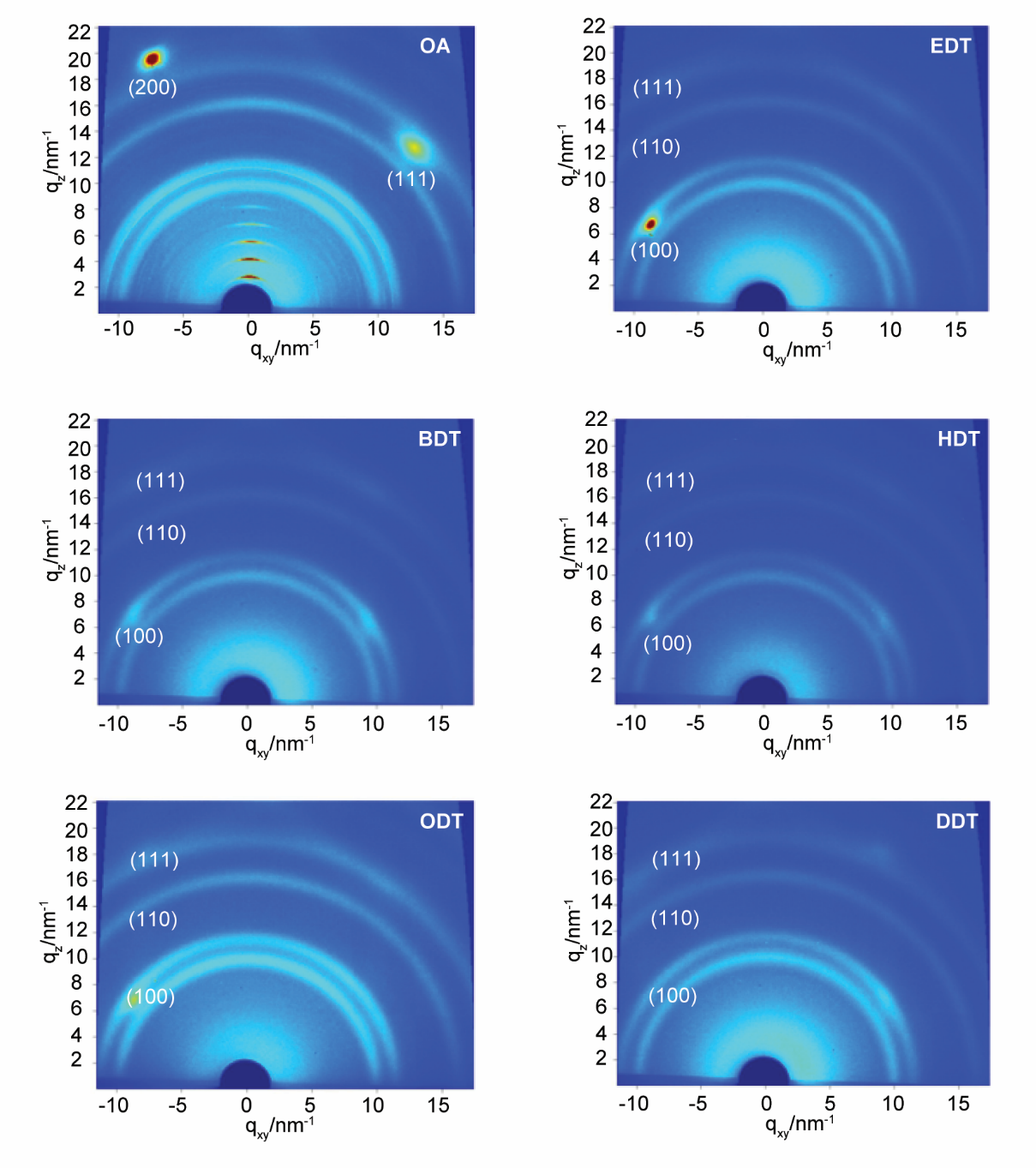


**Fig.** **S6** The GIXRD pattern of PbS QDs films passivated by different alkanedithoil ligands.

The EDT-PbS QDs sample possesses a stronger peak intensity of (100) facet at *φ*~40.5°. This verifies the well-oriented EDT-PbS QDs film, which results in large horizontal leakage electrons. The films treated with BDT, HDT, and ODT also show relatively small peaks of (100) facets at the same azimuthal angle. In comparison, DDT-PbS QDs film shows much more homogeneous intensity distribution over the azimuthal angle. Such a phenomenon is potentially caused by the less energy-preferred exchange of DDT due to its much longer chain length over EDT ^7^.


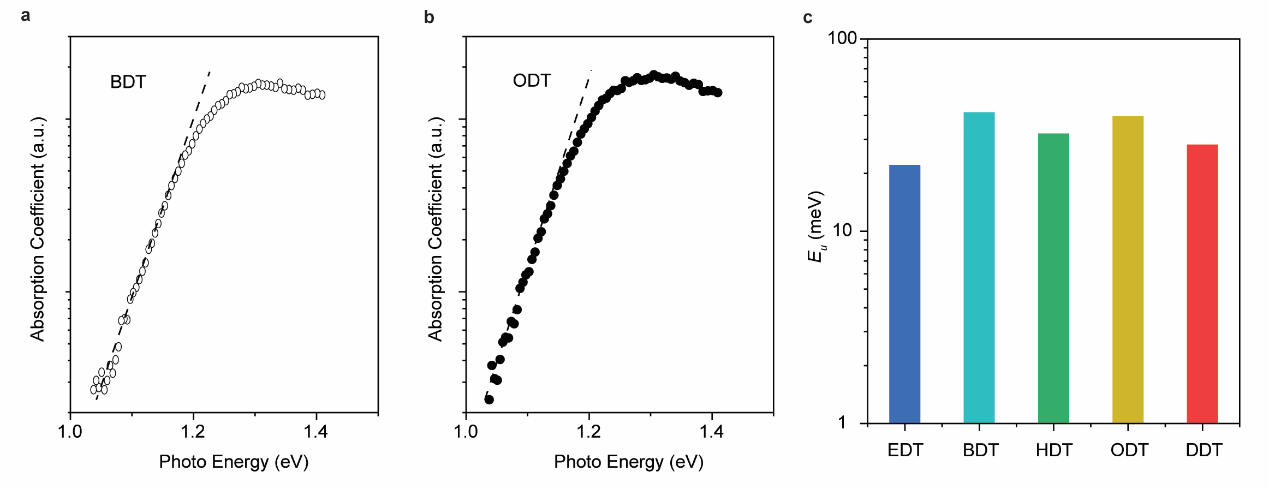


**Fig.** **S7** **a, b** The photothermal deflection spectroscopy (PDS) of BDT-PbS QDs and ODT-PbS QDs. **c** The Urbach energy (*E_u_*) of PbS QDs passivated by different alkanedithiol ligands.

In addition to the PDS data of EDT-PbS QDs, HDT-PbS QDs, and DDT-PbS QDs, the BDT, ODT PDS data is plotted in Fig. S7, with the comparison of Urbach energy for different ligands. By comparing the PDS results of PbS QDs passivated by EDT, BDT, HDT, ODT and DDT, we found that the Urbach energy doesn’t increase dramatically with the ligand length. Medium-length ligands BDT/HDT/ODT show large Urbach energy over 30 meV, while the short EDT and long DDT have relatively low Urbach energy below 30 meV. It could be inferred that longer ligand length in alkanedithiol ligands doesn’t necessarily induce more tail states and indicates the relatively low tail state levels in DDT-PbS QDs, which could partially account for the good performance of DDT-PbS QDs devices in this work.


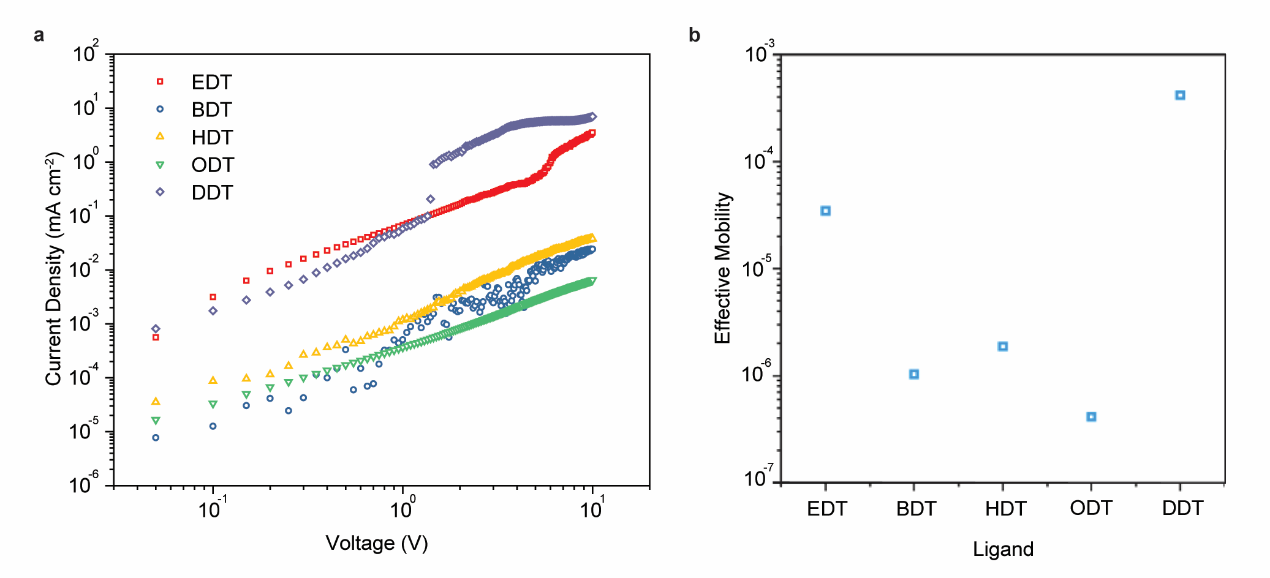


**Fig. S8** **a** The SCLC based on the device structure ITO/IGZO/PbS QDs/ZnO/Al. **b** Extracted effective mobility of PbS QDs with different ligands.

This space charge limited current (SCLC) data supports better vertical transport at the PbS QDs/IGZO interface with DDT ligand passivation. The fitted effective mobility doesn’t show a simple trend according to chain length but shows efficient vertical electron transport for not only very short ligands such as EDT but also for long ligands such as DDT. According to previous studies, the intrinsic carrier mobility of alkanedithiol ligands decreases when ligand length rises from EDT to DDT ^1^, due to increased QDs space. However, effective mobility also reflects the vertical transport between QDs and IGZO films. That is the reason why the effective mobility first drops from EDT to ODT and then rises from ODT to DDT, which indicates there is an additional increment factor in DDT for the effective mobility rise. There is a strong possibility that good vertical transport between DDT/IGZO is this increment.


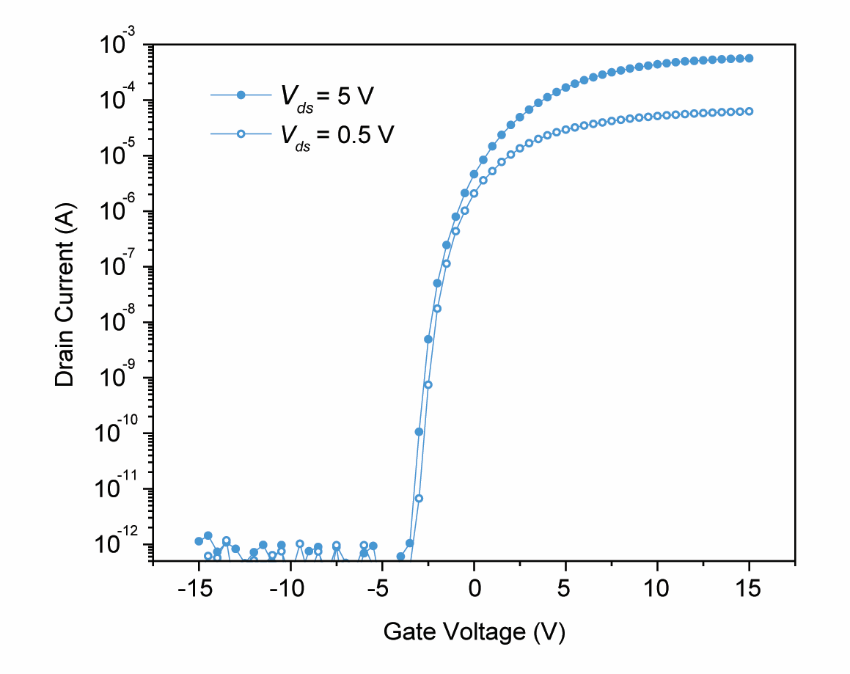


**Fig.** **S9** The *I_d_-V_g_* curve of PbS QDs/IGZO phototransistors passivated by DDT under the dark environment with *V_ds_* = 0.5 V/5 V.

Fig. S9 shows the *I_d_-V_g_* plot of the PbS QDs/IGZO phototransistor with DDT ligand under a dark environment. The on-current of the phototransistor at *V_ds_* bias of 0.5 V and 5 V, is around 60 μA and 500 μA. The off-current is around 1 pA, which is limited by the measurement limit.


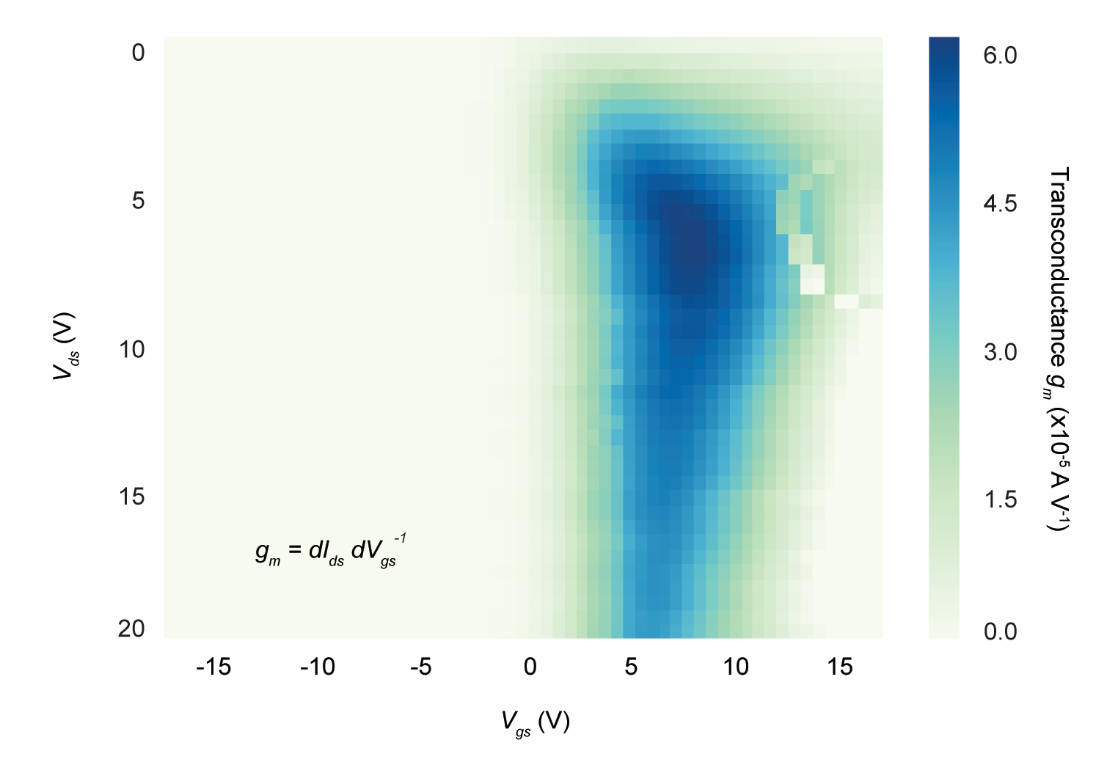


**Fig.** **S10** The mapping of IGZO TFT transconductance *g_m_* versus *V_ds_* from 0 V to 20 V and *V_gs_* from -15 V to 15 V.

The consistency between the mapping of transconductance and responsivity (Fig. 3d) infers the dominance of gate control of responsivity in such phototransistor structures.


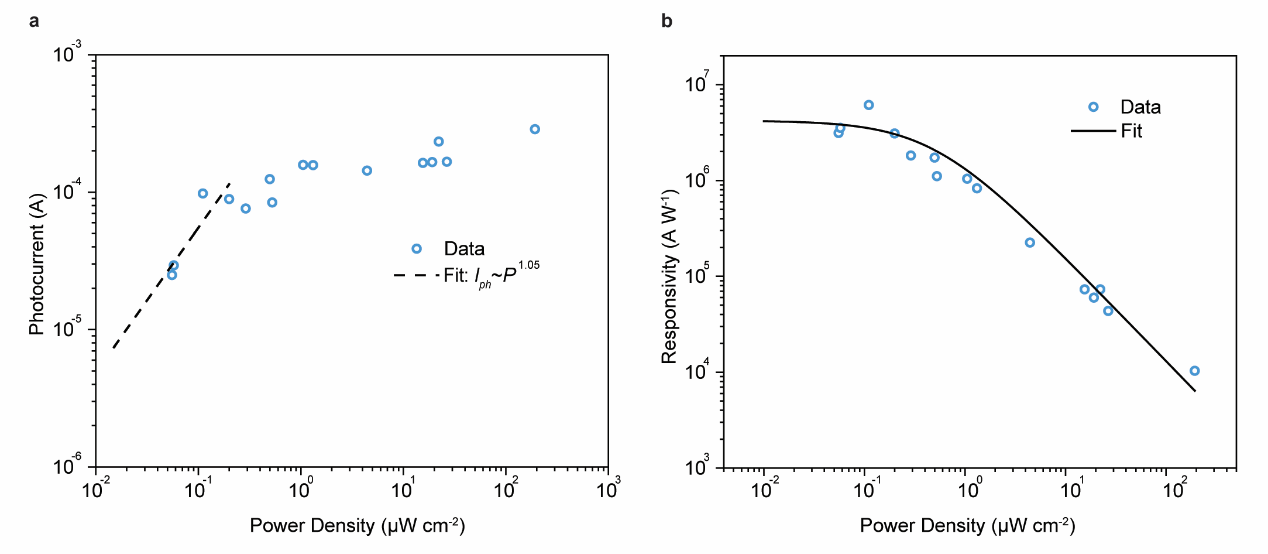


**Fig.** **S11** The power dependence of **a** photocurrent and **b** responsivity for the PbS QDs/IGZO phototransistor with DDT ligand.

The responsivity starts to deteriorate when incident optical power density exceeds 0.1 μW cm^-2^. However, before 0.1 μW cm^-2^, the photocurrent still follows the power’s law. All measurements were conducted using incident light at 850 nm.


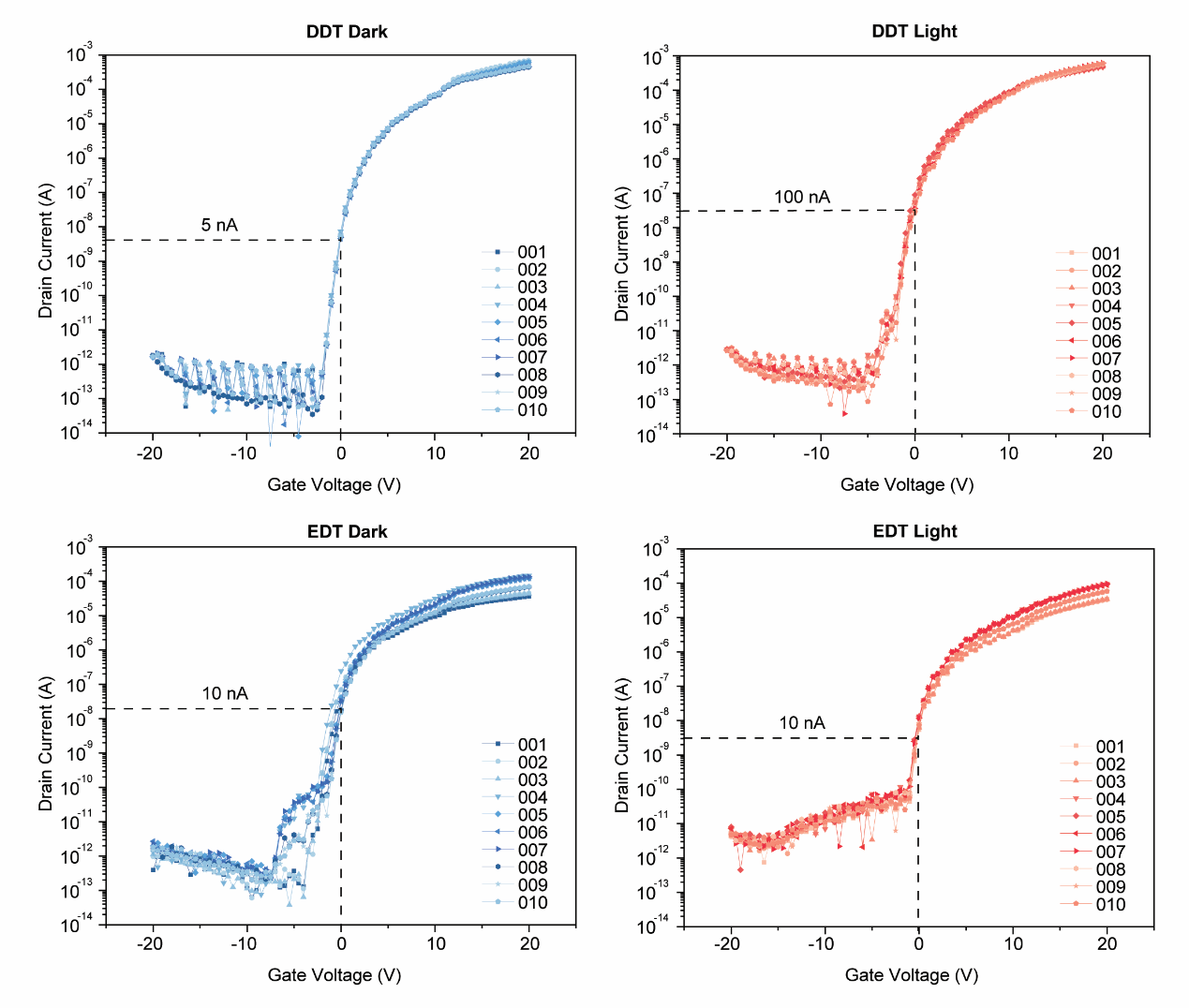


**Fig.** **S12** The collection *I_d_-V_g_* curve of 10 DDT-PbS and 10 EDT-PbS QDs/IGZO phototransistors under both dark and lighting environments. (*V_ds_* = 5 V) The light source adopts white light with an intensity of 1170 μW cm^-2^.

10 DDT and 10 EDT devices were measured in a dark and lighting environment to compare the *I_d_-V_g_* characteristics between EDT and DDT phototransistors and to verify the variation of DDT and EDT ligand exchange processes. As for the *I-V* performance, it is evident that DDT devices not only show higher on-current and better subthreshold behavior, but also show much higher responsivity. To be more specific, EDT devices have a “hump” when the gate voltage is between -10 and 0 V in dark *I-V* while having large leakage current in light *I-V*, which could be induced by the horizontal leakage current as discussed in Fig. 2 and Fig. S5. Also, the EDT devices have a much lower ratio of photocurrent/dark-current, showing much lower responsivity (DDT devices have ~20 times larger light/dark ratio at *V_gs_* = 0 V).

The collection of the IV data infers good consistency and high reproducibility of DDT devices, while the EDT devices show much larger variation for threshold voltage and drain current. Specifically, DDT and EDT devices have threshold voltage shifts below 0.1 V and over 1 V under dark environments, respectively. For both dark and photocurrent, DDT and EDT devices have on-current variation within 20 % and over 60 %, respectively. Such difference in variation indicates the robust ligand exchange processes of DDT. As indicated by GIXRD results (Fig. S6), we infer that EDT with shorter chain lengths might tend to pack PbS QDs in a specific orientation, causing more variation in molecule dynamics and device performance.


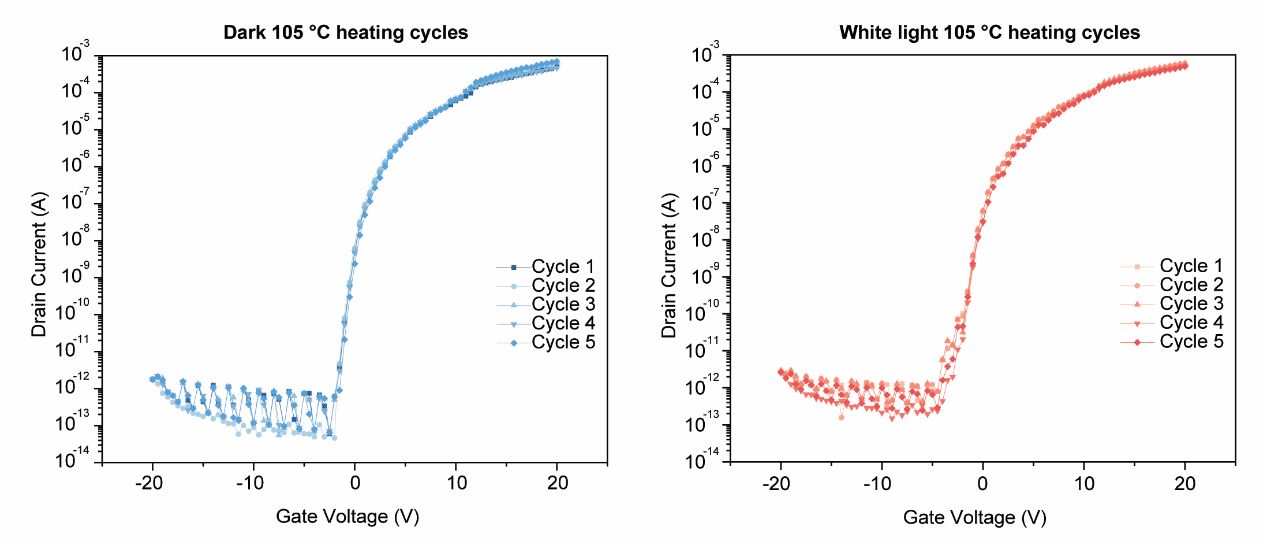
**Fig.** **S13** The thermal cycle stability test (5 cycles) of the DDT-PbS QDs/IGZO phototransistor at 105 °C under the dark and lighting environment. In one thermal cycle, the device was annealed from room temperature to 105 °C and measured for both dark current and photocurrent at 105 °C (measurement ~10 mins), followed by a cooling process to room temperature. This cycle was repeated five times. The light source adopts white light with an intensity of 1170 μW cm^-2^ to cover variation and stability over the visible/near-infrared spectrum.

The thermal stability of the DDT-PbS QDs/IGZO phototransistor was tested following a five-time thermal cycle from room temperature to 105 °C. The device shows a very consistent dark *I-V* curve at each measurement cycle, without a remarkable shift of *V_th_* or degradation of current, which indicates the good thermal stability of the DDT phototransistor at 105 °C. Such stability was demonstrated not only under dark conditions but also under lighting conditions.


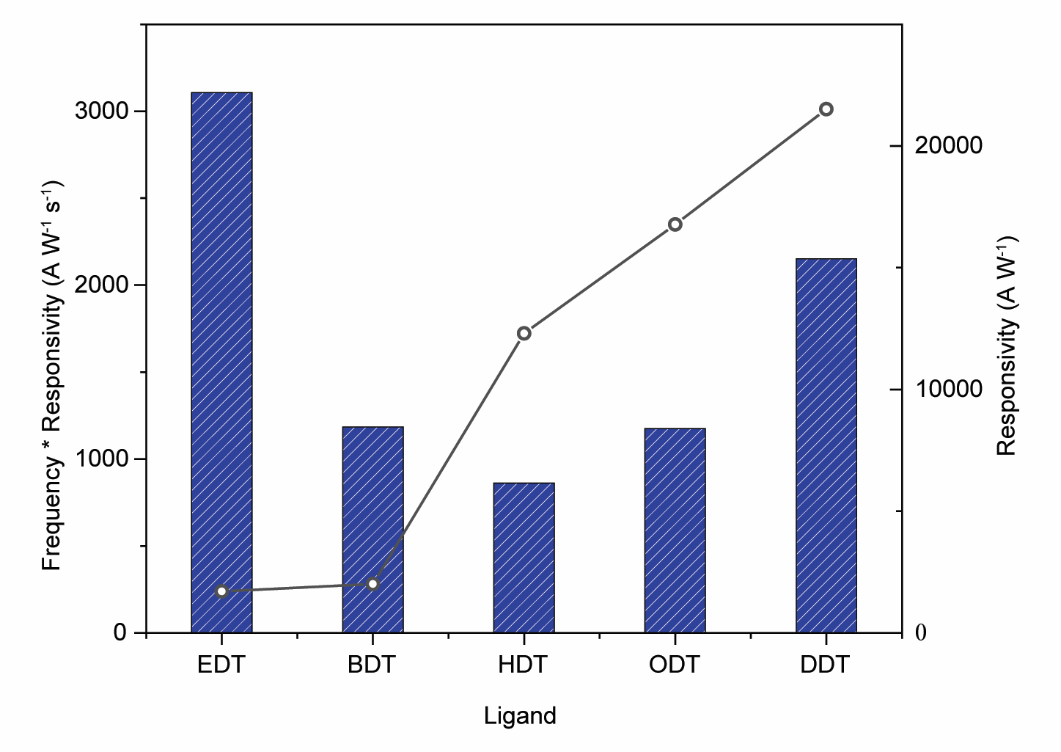


**Fig.** **S14** The product of responsivity and frequency (95 % response) for PbS-QDs/IGZO phototransistors passivated by different ligands.

Since the frequency response is different from EDT to DDT, the responsivity is normalized into the product of responsivity and frequency, where the device has 95 % of the maximum responsivity (according to Fig. S4). This value could be interpreted as the “responsivity per second”. It shows that even with the lowest response frequency, DDT’s normalized responsivity is higher than BDT, HDT, and ODT. Even compared to EDT, DDT has a comparable value over 2000 A W^-1^ s^-1^. The much higher responsivity of the DDT device compared to the EDT device indicates that the DDT device could sense a much slighter signal with longer exposure time, which is suitable for low-dose detection applications.

**Table. S2** Performance comparison of the state-of-the-art PbS phototransistors and the device in this work for the detection of near-infrared light.

| **Channel** | **Photoactive Material** | **Ligand** | **Wavelength (nm)** | **Detectivity (Jones)** | **Responsivity (A/W)** | **Power (pW)** | **Frequency (Hz)** | **Reference** |
| --- | --- | --- | --- | --- | --- | --- | --- | --- |
| Si | PbS QDs | TBAI | 1300 | 1.80E+12 | 3.00E+04 | / | 1.00E+05 | ^8^ |
| IGZO | PbS QDs | EDT | 1000 | 3.00E+13 | 1.00E+06 | 5.97E+00 | 1 | ^9^ |
| PbS QDs | PbS QDs: MoO_3_ | TABI | 1000 | 2.00E+10 | 4.00E+00 | 6.32E+02 | 1.00E+05 | ^10^ |
| Graphene | PbS QDs:Graphene | EDT | / | 7.00E+13 | 1.00E+07 | 0.80E-02 | 10 | ^11^ |
| IGZO | PbS QDs | DDT | 850 | 1.30E+14 | 4.00E+06 | 8.30E+00 | 1 | This Work |

* Industry progress: STMicroelectronics developed a global shutter PbS QDs image sensor at 930 nm detecting wavelength with responsivity ~ 0.4 A W^-1^ ^12^. Since the related industry works are mainly photodiodes instead of phototransistors, we didn’t involve them in the benchmark table.

* tetrabutylammonium iodide: TBAI

**Noise Analysis**

Fig. 3c shows a typical low-frequency noise spectrum of a DDT PbS-QDs/IGZO phototransistor. Before reaching 10 Hz, the noise density gradually decreases with the increase of frequency, showing the behavior of 1/f flicker noise (noise density ~ 1/f^β^) ^13^. Here, the parameter *β* is larger than 1 mainly due to the following reasons. 1) Firstly, the generation-recombination noise is a typical noise in the photodetectors, which have a parabolic noise density spectrum and will increase the effective *β*. 2) Secondly, the low-frequency noise, especially the noise below 10 Hz, could be largely impacted by the environment disturbance and intrinsic equipment/setup noise. This, to some degree, increases the noise level at a lower frequency and thus also results in a higher *β* value. However, even with those factors that could potentially increase the low-frequency noise, as-prepared DDT phototransistors show quite low noise levels enabled by device and measurement optimizations.

**Reference**

1. Zhang, J. et al. Carrier transport in PbS and PbSe QD films measured by photoluminescence quenching. *The Journal of Physical Chemistry C*  ***118***, 16228-16235 (2014**)**.

2. Kim, B.-S. et al. Inorganic-ligand exchanging time effect in PbS quantum dot solar cell. *Applied Physics Letters* ***109****,* (2016).

3. Koole, R. et al. Electronic Coupling and Exciton Energy Transfer in CdTe Quantum-Dot Molecules. *Journal of the American Chemical Society* ***128***, 10436-10441 (2006).

4. Ma, W. et al. Photovoltaic Devices Employing Ternary PbS_x_Se_1-x_ Nanocrystals. *Nano Letters* ***9***, 1699-1703 (2009).

5. Liu, M. et al. Hybrid organic–inorganic inks flatten the energy landscape in colloidal quantum dot solids. *Nature Materials* ***16***, 258-263 (2017).

6. Septianto, R. D. et al. Enabling metallic behaviour in two-dimensional superlattice of semiconductor colloidal quantum dots. *Nature Communications* ***14***, 2670 (2023).

7. Hou, B. et al. Evolution of local structural motifs in colloidal quantum dot semiconductor nanocrystals leading to nanofaceting. *Nano Letters* ***23***, 2277-2286 (2023).

8. Adinolfi, V. & Sargent, E. H. Photovoltage field-effect transistors. *Nature* ***542***, 324-327 (2017).

9. Hwang, D. K. et al. Ultrasensitive PbS quantum-dot-sensitized InGaZnO hybrid photoinverter for near-infrared detection and imaging with high photogain. *NPG Asia Materials* ***8***, e233-e233 (2016).

10. Adinolfi, V. et al. Photojunction field-effect transistor based on a colloidal quantum dot absorber channel layer. *ACS nano* ***9***, 356-362 (2015).

11. Goossens, S. et al. Broadband image sensor array based on graphene–CMOS integration. *Nature Photonics* ***11***, 366-371 (2017).

12. Steckel, J. S. et al. 1.62*μ*m Global Shutter Quantum Dot Image Sensor Optimized for Near and Shortwave Infrared. *IEEE International Electron Devices Meeting (IEDM)* 23.4.1-23.4.4 (2021).

13. Theodorou, C. G. et al. Origin of low-frequency noise in the low drain current range of bottom-gate amorphous IGZO thin-film transistors. *IEEE electron device letters* ***32****,* 898-900 (2011).
